# The Precentral Gyrus Contributions to the Early Time-Course of Grapheme-to-Phoneme Conversion

**DOI:** 10.1162/nol_a_00047

**Published:** 2022-02-10

**Authors:** Erik Kaestner, Xiaojing Wu, Daniel Friedman, Patricia Dugan, Orrin Devinsky, Chad Carlson, Werner Doyle, Thomas Thesen, Eric Halgren

**Affiliations:** Center for Multimodal Imaging and Genetics, University of California, San Diego, USA; Department of Neurology, NYU Langone School of Medicine, New York, USA; Department of Neurology, Medical College of Wisconsin, Milwaukee, USA; Department of Neurosurgery, NYU Langone School of Medicine, New York, USA; Department of Neurosciences, University of California at San Diego, La Jolla, USA; Department of Radiology, University of California at San Diego, La Jolla, USA

**Keywords:** electrocorticography, reading, graphemes, phonemes, audiovisual integration

## Abstract

As part of silent reading models, visual orthographic information is transduced into an auditory phonological code in a process of grapheme-to-phoneme conversion (GPC). This process is often identified with lateral temporal-parietal regions associated with auditory phoneme encoding. However, the role of articulatory phonemic representations and the precentral gyrus in GPC is ambiguous. Though the precentral gyrus is implicated in many functional MRI studies of reading, it is not clear if the time course of activity in this region is consistent with the precentral gyrus being involved in GPC. We recorded cortical electrophysiology during a bimodal match/mismatch task from eight patients with perisylvian subdural electrodes to examine the time course of neural activity during a task that necessitated GPC. Patients made a match/mismatch decision between a 3-letter string and the following auditory bi-phoneme. We characterized the distribution and timing of evoked broadband high gamma (70–170 Hz) as well as phase-locking between electrodes. The precentral gyrus emerged with a high concentration of broadband high gamma responses to visual and auditory language as well as mismatch effects. The pars opercularis, supramarginal gyrus, and superior temporal gyrus were also involved. The precentral gyrus showed strong phase-locking with the caudal fusiform gyrus during letter-string presentation and with surrounding perisylvian cortex during the bimodal visual-auditory comparison period. These findings hint at a role for precentral cortex in transducing visual into auditory codes during silent reading.

## INTRODUCTION

The interaction of an initially auditory-only perisylvian language network and a later developing reading network has been studied since the late 1800s (Dejerine, 1892). Decades of intricate behavioral work (Frost, 1998; Rastle & Brysbaert, 2006) combined with lesion studies (Coltheart, 1980; Geschwind, 1974) led to the development of cognitive (Coltheart et al., 2001; Diependaele et al., 2010; Harm & Seidenberg, 2004; Perry et al., 2007) and neuroanatomical (Carreiras et al., 2014; Fiez & Petersen, 1998; Jobard et al., 2003; Price, 2012; Taylor et al., 2013) models which include interactive processing of both the graphemic and phonological information contained in visual text. This necessitates the transduction of graphemic into auditory phonological codes, followed by a period of lexical-semantic processing in which visual and auditory processing interacts and influences one another. But basic questions of how a visual stimulus is transduced into an auditory code, known as *grapheme-to-phoneme conversion* (GPC), remain unresolved. GPC has been associated with both phonological encoding, classically located with Wernicke’s area in lateral temporal-parietal regions, and phonological articulation, associated with frontal regions. However, the possible role of the articulatory phonological representations in the precentral gyrus in GPC remains ambiguous.

Early lesion studies focused attention on temporal-parietal regions as the entryway for visual text into the wider auditory perisylvian lexical-semantic network via GPC (Geschwind, 1974). Blood oxygen level-dependent (BOLD) fMRI studies provided early support for this localization, with phonological decisions evoking greater activation than other decisions in the angular gyrus (Binder et al., 2005; Booth, 2002; McDermott et al., 2003) with several neuroanatomical models of reading subsequently incorporating this locus (Carreiras et al., 2014; Taylor et al., 2013). Also implicated is the nearby superior temporal gyrus (STG): It is a critical processing area for auditory phonemes (Leonard et al., 2015; Mesgarani et al., 2014; Travis et al., 2013) with phonologically related activity also evoked by visual language stimuli (Booth, 2002). Intracranial electrophysiology studies have also found overlapping (Perrone-Bertolotti et al., 2012) and correlated (Chan et al., 2014) activity to auditory and visual language in the STG.

However, early psychological theory associated GPC with articulatory phonological cognitive operations (Allport, 1979; Barron & Baron, 1977; Burani et al., 1991; Klapp, 1971; Kleiman, 1975; Peterson & Johnson, 1971), presumably in articulatory motor cortex. This was based on data from the articulatory suppression paradigm in which participants repeated a nonsense phrase to occupy the articulatory cognitive operations while performing a reading task. Results showed suppressed phonological effects (Barron & Baron, 1977; Burani et al., 1991; Kleiman, 1975; Sun & Peperkamp, 2016) but not if mouth movements were non-articulatory (Burani et al., 1991) or if the words were presented auditorily (Peterson & Johnson, 1971). A variety of BOLD neuroimaging and lesion studies support precentral involvement in silent reading. Masked phonological priming evokes activity in the left precentral gyrus even when the words are not consciously perceived (Dehaene et al., 2001). Studies also find greater activity in the precentral gyrus when making phonological judgements for visual words (Price et al., 1997; Yen et al., 2019), with differential activation based on spelling-sound consistency (Fiez et al., 1999) and difficultly of GPC (Binder et al., 2005). A perirolandic lesion reduced a patient’s ability to make phonological judgements about words (Vallar et al., 1997). Another patient with a perirolandic lesion similarly retained comprehension of visual words but was unable to make rhyming judgements or manipulate pseudowords (Vallar & Cappa, 1987). In a patient with phonological alexia and agraphia, increased activation in the precentral gyrus was observed during successful cognitive rehabilitation of phonological processing (DeMarco et al., 2018). Finally, in transcranial magnetic stimulation, greater excitability in motor cortex during GPC is reported (Lauro et al., 2020). The neighboring inferior frontal gyrus cortical region pars opercularis has been similarly tied to phonological effects (Cornelissen et al., 2009; Pammer et al., 2004; Wheat et al., 2010). Therefore, in addition to the lateral temporal-parietal contributions to GPC, there is a strong case that the precentral gyrus is involved in GPC as well.

### The Present Study

Here, we test the theory that the precentral gyrus is a contributor to GPC using a silent match/mismatch task in which a 3-letter string containing sublexical phonological information (e.g., “GUH”) was presented first, followed by auditory presentation of an auditory bi-phoneme. During this task, intracranial electrophysiological (iEEG) activity was recorded from the cortex. In cognitive models of reading, activation of letter/grapheme representations begins first, and then is passed along to the graphemes’ phonological analogues by GPC (Coltheart et al., 2001; Diependaele et al., 2010; Harm & Seidenberg, 2004). By using a bimodal task with iEEG, which possesses high spatial-temporal resolution, we will observe the evolution of the putative phonological-based processing during visual language encoding and then how this activity is modified by incoming phonological information derived from auditory language encoding. We will assess three hypotheses: (1) that a task focused on GPC will evoke visual language activity in the precentral gyrus, (2) that this visual language evoked activity occurs at a time consistent with precentral gyrus contributions to GPC, and (3) whether visual language evoked activity is overlapping with auditory language evoked activity in the precentral gyrus.

The first hypothesis will be addressed by examining the distribution of increased activity to linguistic stimuli compared to their sensory controls in perisylvian regions. Our hypothesis predicts that we will identify activity in the precentral gyrus at least as often as in the surrounding frontal (e.g., pars opercularis) and temporal-parietal (e.g., STG and supramarginal) regions which are highlighted in neurobiological models of reading (Carreiras et al., 2014; Fiez & Petersen, 1998; Jobard et al., 2003; Price, 2012; Taylor et al., 2013), providing evidence that the precentral gyrus is also active during GPC. Further, we will examine whether the precentral gyrus demonstrates connectivity with the ventral visual language processing regions such as the fusiform (Dehaene & Cohen, 2011; Lochy et al., 2018; Vinckier et al., 2007) as well as the surrounding perisylvian regions implicated in visual language processing.

For the second hypothesis, we will characterize the time window of the observed activity in the precentral gyrus. As mentioned, cognitive models of reading start with activation of letter/grapheme representations followed by activation of phonemes via GPC (Coltheart et al., 2001; Diependaele et al., 2010; Harm & Seidenberg, 2004). Neurobiological evidence confirms this sequence. Letter/grapheme encoding occurs in posterior occipital-temporal regions beginning at ∼160–180 ms (Allison et al., 1994, 1999; Hirshorn et al., 2016; Thesen et al., 2012). This is followed by widespread onset of visual language evoked activity across large portions of the brain at around the same time (Halgren, 1990). For GPC, extracranial electrophysiology identifies visual language evoked phonological effects beginning during a similar time window of ∼250–350 ms (Rugg, 1984; Grainger et al., 2006; Holcomb & Anderson, 1993). Therefore, language evoked activity in the precentral gyrus during this time window would be consistent with GPC (a question the spatial-temporal precision of iEEG is well-placed to observe). Further, through network-level analyses we can examine whether the precentral gyrus is significantly coupled with occipital-temporal regions during this time window. Noninvasive imaging studies of resting-state connectivity (Stevens et al., 2017) and diffusion tensor imaging (Bouhali et al., 2014) demonstrate that the precentral gyrus has connectivity with these occipital-temporal regions, but they lack the temporal specificity to understand when this connectivity may occur during visual language processing.

For the third hypothesis, we will seek to observe whether perisylvian visual language processing is overlapping with auditory language processing in the same areas. Extracranial EEG evidence demonstrates that visually presented language primes auditorily presented language, recorded over broad brain regions within several 100 ms of onset (Holcomb et al., 2005; Kiyonaga et al., 2007). Using our match/mismatch task and the increased spatial precision of iEEG, we will assess whether we can detect specific cortical patches which show evidence of auditory phonological representations being primed by visual phonological representations. Second, using this bimodal task will allow us to observe whether phonological representations for auditorily encoded phonemes in the STG (Mesgarani et al., 2014) and the precentral gyrus (Cheung et al., 2016) are also activated by visual language stimuli. If the phonological representations activated during visual language processing are the same phonological representations activated during auditory language processing, we will be able to observe both overlap (i.e., visual and auditory effects in the same electrode) and priming (i.e., differences in activity between matching and mismatching visual/auditory phonemes in the same electrode) of these phonological representations. Previous studies have found evidence that visually and auditorily evoked language activity overlap in the STG when presented at separate times (Chan et al., 2014; Perrone-Bertolotti et al., 2012), but the relationship is unknown for the precentral gyrus. Further, reports of phoneme-specific activity in the STG (Mesgarani et al., 2014) raise the question of whether GPC phoneme-specific activity (i.e., letter-specific activity) can be identified. Taken together, these three hypotheses will inform whether visual language evoked activity in the precentral gyrus during silent visual language encoding is consistent with contributions to GPC.

## MATERIALS AND METHODS

### Participants and Recordings

Electrocorticographic recordings were obtained from 8 patients (5 males, mean age 35.6, age range 17–56; Table 1) undergoing intracranial EEG monitoring as part of treatment for pharmacologically resistant epilepsy. All procedures were approved by the Institutional Review Board at New York University, and written informed consent was obtained from all participants. Electrode placement was determined by clinical criteria to identify seizure activity and eloquent tissue. Each patient was implanted with subdural platinum-iridium electrode arrays embedded in silastic sheets (AdTech Medical Instrument Corp.). Data included arrays of grids (8 × 8 contacts) and strips (1 × 4 to 1 × 12 contacts). Contacts had a diameter of 4 mm with 2.3 mm exposure. Center-to-center spacing between contacts was 10 mm for grids and 5 mm for microgrids. Recordings were acquired using a NicoletOne EEG system (https://neuro.natus.com/) sampled at 512 Hz and bandpass filtered between 0.5 and 250 Hz. In total, there were 5 implantations focused on the left hemisphere and 3 implantations focused on the right hemisphere. Patient language lateralization based on the Wada procedure is noted in Table 1 when it is available, with all patients with a Wada showing left lateralization. Three patients (P4, P5, and P6) did not have Wada information available; however, all were right-handed and therefore believed to have typical (i.e., left) language lateralization.

**Table 1.** Patient clinical information, neuropsychological, and task performance

	**Age**	**Onset**	**Sex**	**Hand**	**Wada**	**Implantation**	**VCI**	**POI**	**WMI**	**PSI**	**Match correct**	**Mismatch correct**	**Match RT (ms)**	**Mismatch RT (ms)**
**P1**	36	12	F	R	L	L	102	113	114	102	95%	98%	588	604
**P2**	47	35	M	R	L	L	72	102	92	77	84%	86%	704	740
**P3**	24	22	F	R	L	R	100	84	83	86	81%	95%	601	595
**P4**	56	30	M	R	–	R	–	–	–	–	94%	84%	611	746
**P5**	25	0.1	M	R	–	R	110	100	83	94	91%	98%	587	640
**P6**	26	14	M	R	–	L	–	–	–	–	86%	88%	755	757
**P7**	54	35	F	R	L	L	96	102	86	94	94%	92%	738	719
**P8**	17	13	M	R	L	L	91	92	89	86	89%	98%	698	627

*Note*. RT = response time; VCI = Verbal Comprehension Index; WMI = Working Memory Index; POI = Perceptual Organization Index; PSI = Processing Speed Index.

### Electrode Localization

Electrode localization was done through co-registration of pre- and postimplant MRI images, followed by manual and automatic localization of electrodes (Yang et al., 2012). Coordinates were co-registered to a standard MNI template and anatomical parcellations were determined using a Desikan atlas (Desikan et al., 2006). For display purposes the atlas was slightly modified by splitting long gyri into 3 equal segments, either inferior/middle/superior (precentral, post-central parcellations) or caudal/middle/rostral (fusiform, inferior temporal, middle temporal, super temporal, middle-frontal, superior-frontal parcellations). Three-dimensional reconstructions of cortical surfaces were created using FreeSurfer (Dale et al., 1999). Electrode localization into a parcellation region was performed in each subject’s native brain space. Average electrode locations, used only for display purposes, were obtained using FreeSurfer surface-to-surface calculations with the fsaverage brain. Regions with <5 electrodes were excluded from analysis.

### Task Design

**Figure 1.**
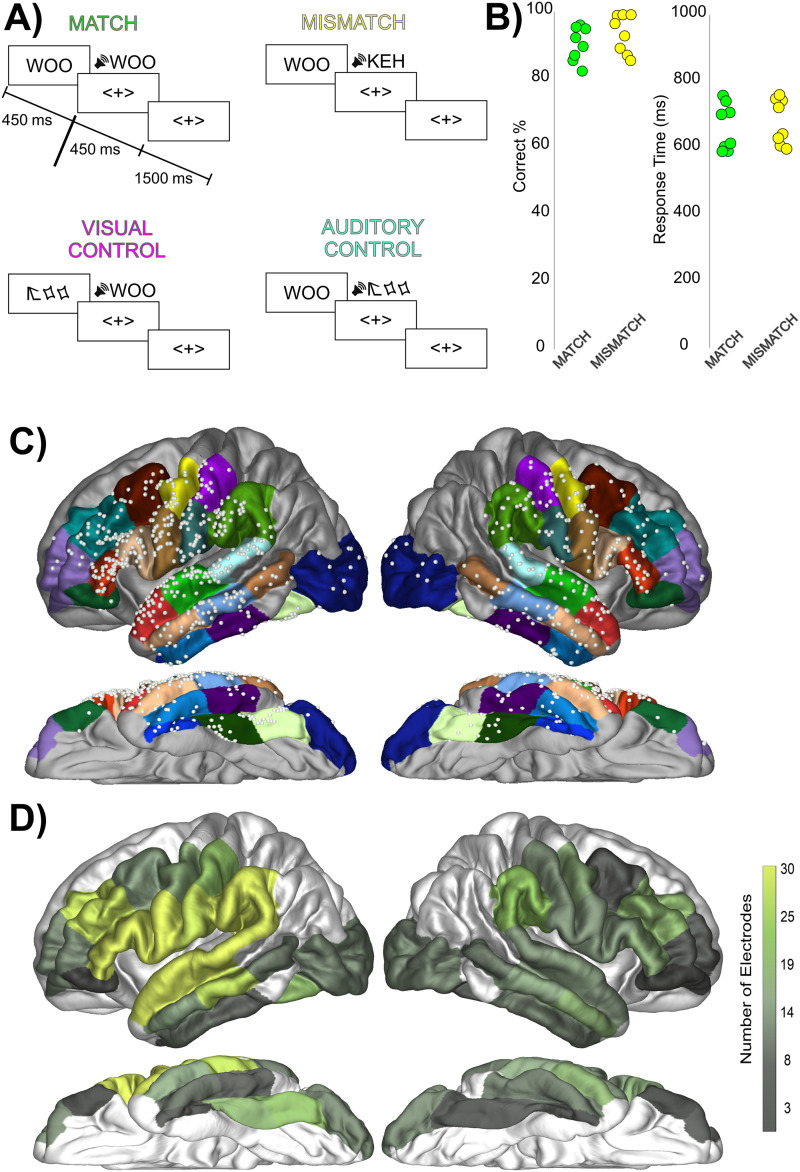
Task design, performance, and electrode coverage. (A) The sequence of stimulus presentation in the four trial types. (B) Patient performance on the Match and Mismatch trials as expressed by percentage correct and response time, demonstrating similar performance across the two trial types. Each dot represents the performance of one patient. (C) Electrode coverage highlighting the coverage of perisylvian electrodes. Electrode coverage across the included regions of interest (ROIs) presented on an average brain for illustration purposes. Colors on brain highlight the ROIs involved in the study from the Desikan atlas. Grey regions were not included in analyses due to lack of electrode coverage in ROIs. (D) Electrode coverage expressed as the total number of electrodes within each ROI.

Figure 1A displays a schematic of the task. Patients performed a silent match/mismatch decision between a 3-letter string (e.g., “GUH”) and a subsequently following bi-phoneme. Bi-phonemes were created by recording both a male and a female speaker with all stimuli volume normalized and length normalized to 450 ms in postprocessing using Adobe Audition. All stimuli were in consonant-vowel order. Both the letter-string (onset 0 ms) and bi-phoneme (onset 450 ms) were presented for 450 ms. The visual stimulus was presented first, replacing a 3-symbol fixation (<X>) for 450 ms, then immediately returning to the fixation. The bi-phoneme was played next, which lasted for 450 ms, followed by a response period. In total, 1,000 ms from bi-phoneme onset were allowed for a participant response. Stimulus onset asynchrony was varied between 700 and 1,000 ms randomly.

This was a silent task with participants responding with their hand, ipsilateral to the hemisphere being recorded from to avoid hand-motor movement activity contaminating the recordings. The vocal silence of the task ensured that any activity observed in putative articulatory cortex was sub-articulatory and not related to overt motor movement.

In total, four types of trials were presented. The first two trial types were Match and Mismatch, in which the letter-string and the bi-phoneme either phonetically matched or did not. The final two trial types provided visual and auditory sensory controls. In Visual Control trials a 3-symbol false-font was displayed, followed by a normal bi-phoneme. These false-font stimuli share the same basic visual sensory features matched to regular letters. Each false-font character was matched to a real letter in the English alphabet in size, number of strokes, total line length, and curvature (for example see Figure 1A; Thesen et al., 2012). In Auditory Control trials the normal letter-string was displayed followed by a noise-vocoded stimulus. Noise-vocoded stimuli preserve temporal envelope cues in broad frequency bands but restrict the listener to degraded information on the distribution of spectral energy. Therefore, temporal and amplitude cues of speech were preserved in each spectral band, providing a control for the sensory characteristics of speech, but the spectral detail within each band was degraded. A study of noise-vocoded speech processing using iEEG found that the number of bands determined how widespread processing of the stimuli was in perisylvian regions. At lower numbers of bands (1–2 bands), speech was unintelligible and evidence of processing was largely restricted to Heschl’s gyrus. However, at higher numbers of bands (≥3 bands for good performers, at least >4 bands for bad performers), intelligibility increased above chance and activity was found in regions such as the STG, though activity evoked by noise-vocoded stimuli was still less than clear speech (Nourski et al., 2019). Because we were interested in processing beyond Heschl’s gyrus in regions such as the superior temporal and precentral gyri, we chose to have a higher number of bands (i.e., to include some intelligibility in our noise-vocoded stimuli). Therefore, noise-vocoded stimuli were created by taking the existing bi-phoneme stimuli and creating a 6-band stimulus in which white-noise was multiplied by power in each of the bands to create a matched set of auditory stimuli with identical time-varying spectral acoustics (Chan et al., 2014; Chen & Loizou, 2011; Horowitz, 2014; Souza & Rosen, 2009; Travis et al., 2013). On both types of control trials, participants were instructed to respond with a Mismatch response. In total, there were 768 trials, with 192 of each of the four trial types. These trials were broken down into 3 runs. Within each run, letter-string/bi-phoneme stimuli were created by crossing 4 consonants and 4 vowels (i.e., 16 bi-phoneme combinations per run) to facilitate balanced presentations of each letter and phoneme.

### Data Processing

Data were preprocessed using MATLAB (MathWorks), the Fieldtrip toolbox (Oostenveld et al., 2011), and custom scripts. We used an average subtraction reference for each patient to remove global artifacts and noise, followed by a bandstop around line-noise and its harmonics (60, 120, 180 Hz). Data were epoched to the onset of the letter-string, from −1,500 to 2,500 ms, to avoid epoch-related edge artifacts introduced by converting from the time domain to the frequency domain. Temporal padding was removed at the end of preprocessing for finalized −500 to 1,500 ms epochs. To calculate broadband high gamma (BHG), epochs were transformed to the time–frequency domain using the wavelet transform from 70 to 170 Hz in 10 Hz increments. Constant temporal and frequency resolution across target frequencies were obtained by adjusting the wavelet widths according to the target frequency. The wavelet widths increase linearly from 14 to 38 as frequency increased from 70 to 170 Hz, resulting in a constant temporal resolution with a standard deviation of 16 ms and frequency resolution of 10 Hz. For each epoch, spectral power was calculated from the wavelet spectra, normalized by the inverse square frequency to adjust for the rapid drop-off in the EEG power spectrum with frequency, and averaged from 70 to 170 Hz, excluding line noise harmonics. This data was smoothed by a moving window matching the temporal characteristics of the wavelet (i.e., a normal distribution with 16 ms standard deviation). Each trial epoch was demeaned with a baseline from −250 to 0 ms. Trials containing artifacts were identified by amplitude and variance, visually inspected for artifacts, and removed from further analysis.

### Analysis

#### Behavior

We compared patient performance and response speed on Match and Mismatch trials with a *t* test to gauge if electrophysiological differences between these two trial types could be attributed to differences in difficulty.

#### Task-modulation

Our first goal was to identify electrodes that were responsive and modulated by our task manipulations. Electrodes that had significantly increased activity from a baseline of 0 to any of the four trial types between 50 and 900 ms were identified using a timepoint-by-timepoint *t* test corrected for temporal false-discovery rate at *p* < 0.05 (Benjamini & Hochberg, 1995). Next, a one-way ANOVA was run between the four trial types from 50–900 ms at *p* < 0.01 temporally corrected using a bootstrapped shuffling of trial identity 1,000 times (Maris & Oostenveld, 2007). Only electrodes which were significant in both these tests (i.e., with a significant increase from baseline in BHG and a significant difference between trial types during this increase) were included in further analysis. We refer to these as *Task-Modulated electrodes*.

#### Language-preference

Next we sought to understand if the evoked activity was related to language processing by comparing visual and auditory language to their sensory controls. Task-Modulated electrodes were assessed for whether they were responding preferentially to either visual or auditory linguistic stimuli as evidenced by an increased response to letter-strings relative to false-font stimuli from 50–450 ms (*Text-Preference*) or an increased response to bi-phonemes relative to noise-vocoded stimuli from 450–900 ms (*Phoneme-Preference*). ANOVAs were run timepoint-by-timepoint, once again corrected using the bootstrapped shuffling method. The Task Modulation ANOVA results were used to mask significant Text-Preference and Phoneme-Preference time-periods to ensure differences found between stimulus types were part of the originally identified Task-Modulated temporal period.

#### Individual letter and phoneme identity sensitivity

A key question is the location of the cortical representation of sublexical linguistic units for letters and the overlap of these letter representations with phoneme representations. An area which contains such representations would be expected to have differential neural responses based on letter/phoneme identity. To assess such sublexical representations, a 1-way ANOVA was run timepoint-by-timepoint on Task-Modulated electrodes between consonant identity for either letter-strings from 0–450 ms (*Letter-Sensitive*) or bi-phonemes from 450–900 ms (*Phoneme-Sensitive*), temporally corrected using the bootstrapped shuffling method. The “Task Modulated” ANOVA results were used to mask significant time-periods.

#### Mismatch effect

Successfully performing our match/mismatch task necessitates encoding phonemes presented both visually and auditorily. If the same phonological representations are used by both sensory modalities (i.e., strong overlap of phonological processing), then cortical patches containing these representations should show differential processing depending on whether the visual and auditory phonemes match or mismatch, due to repetition priming. In BHG, repetition priming (i.e., re-encoding a recently encoded stimulus) evokes reduced power (McDonald et al., 2010). This is likely because re-encoding a recently encoded stimulus evokes less neuronal activity than the initial encoding (Gotts et al., 2012). Mismatch-sensitive electrodes were therefore defined as having a larger BHG response to mismatch trials (i.e., non-primed trials) than to matched trials (i.e., primed trials) during presentation of the bi-phoneme (i.e., from 500–900 ms) identified using a 1-way ANOVA temporally corrected using the bootstrapped shuffling method (*Mismatch*). The Task Modulated ANOVA results were used to mask significant time-periods.

#### Preference for degraded speech

Behavioral studies have demonstrated text can improve the encoding of degraded speech, presumably through GPC (Frost, 1991; Frost & Kampf, 1993). To identify areas that may participate in this process, we sought to investigate how noise-vocoded preference overlaps with Text-Preference by identifying electrodes with greater responses to noise-vocoded stimuli versus bi-phonemes from 500–900 ms (*Noise-Preference*), using the same procedure detailed for Phoneme-Preference electrodes. The Task Modulated ANOVA results were used to mask significant time-periods.

### Overlap Comparisons

We also sought to characterize whether visual and auditory effects within a region were observed in separate or in overlapping electrodes. This was operationalized as an examination of the overlap between Text-Preference effects and auditory effects (Phoneme-Preference, Mismatch, Noise-Preference) using the binomial test of overlap percentages compared to chance. Because overlap within a region could happen by chance, we took the percentage of Text-Selective electrodes within a parcellation region as the baseline chance. For example, in the Precentral parcellation, 16 out of 54 electrodes (∼30%) displayed Text-Preference effects, and therefore, randomly distributed auditory effects in this same region would be expected to overlap with 30% of the Text-Preference effects by chance. A significant (i.e., *p* < 0.05 on the binomial test) increase in overlap above this baseline percentage would be evidence of overlap. This comparison was made across all included electrodes across a hemisphere as well as within the regions of interest.

### Regional Comparisons

Comparisons between regions are difficult in iEEG due to sparse coverage with variation due to clinical considerations. However, studies with large numbers of patients note that responses occur in similar regions relative to neuroanatomical landmarks (Ojemann et al., 1989). Our study made use of nonparametric statistics to compare both proportion of electrodes and timing of effect onsets grouped into the broad Desikan atlas (Desikan et al., 2006) parcellations.

To compare distribution across putative articulatory or putative encoding cortex we performed planned a priori comparisons between the precentral parcellation and the STG, supramarginal, and pars opercularis parcellations. A Fisher’s Exact test was used to compare proportions across regions. With three comparisons (i.e., precentral gyrus versus STG, supramarginal gyrus, and pars opercularis), the Bonferroni corrected *p* value threshold was *p* < 0.016. We also compared effect onset timings between regions using a Ranksum nonparametric test, when possible. The effect onset was defined as the first timepoint displaying a significant effect after correction for temporal multiple comparisons. The variable number of effects per parcellation does cause differences in power between comparisons; for example, a critical question is the timing of effects in the precentral gyrus versus the STG and pars opercularis, but while the precentral gyrus had 16 Text-Preference electrodes and the pars opercularis had 7, the STG contained only 3. However, despite these difficulties some temporal regularities emerged. These timing analyses will mirror the a priori structure of the regional distribution analyses and share their *p* value corrections.

### Broadband High Gamma Amplitude

Broadband high gamma amplitude gives an indirect measure of the aggregate surrounding neuronal population firing (Ray et al., 2008). To understand whether amplitude differed between regions, we calculated max amplitude of language trials for letter-string presentation and bi-phoneme presentation within Task-Modulated electrodes. To assess amplitude the average waveform for language was *z* scored relative to its baseline (the same time window as used for baselining in data analysis). Then the highest value was found for each electrode for the letter-string time window (0–450 ms) and the bi-phoneme time window (450–900 ms).

### Connectivity

To test for a putative network between the electrodes displaying effects of interest, we used *phase-locking value* (PLV) calculated pairwise between electrodes as described in Lachaux et al. (1999). PLV measures the consistency of the relative phase of frequencies within the electrophysiological signal. High PLV indicates consistent synchronization of the synaptic currents in pyramidal apical dendrites between the cortical locations underlying the intracranial sensors. For this study we measured frequencies from 4 Hz to 12 Hz. This frequency range was chosen based on a prior paper which found strong phase locking in this frequency range within the reading network (Thesen et al., 2012). Neural activity in this lower frequency range is associated with feedforward and feedback activity across coordinating neural networks during visual language processing (Halgren et al., 2015). To understand whether the PLV values we obtained in Task-Modulated electrodes were greater than would be expected by random chance, we created a distribution of baseline PLV values for each individual patient. This distribution was obtained by taking the max PLV value from a baseline period (−200–0 ms) for all electrode pairs within a patient. A PLV value in the task window for the Task-Modulated electrodes of interest (100–1,300 ms) was considered significant (i.e., greater than would be expected by chance) if it was *p* < 0.00005 based on the subject’s own baseline PLV distribution.

## RESULTS

### Behavior

Average proportion correct for Match (89%) and Mismatch (92%) trials was not significantly different (*p* > 0.05; Figure 1B). Likewise, response time for Match (660 ms) and Mismatch (678 ms) trials was not significantly different (*p* > 0.05). Therefore, there is no behavioral evidence for difficulty differences in performing either judgement. For the control trials, behavioral performance was similar to other trial types for False-Font (94%) and Noise-Vocoded (92%) trials.

### Task-Modulation Effect Distribution

In discussing results for the following sections, we will report results during the letter-string presentation and/or during bi-phoneme presentation. Results during the letter-string presentation relate to the first hypothesis that GPC evokes activity in the precentral gyrus and the second hypothesis that the timing of this activity will be consistent with precentral gyrus contributions to GPC. Results during the bi-phoneme presentation will relate to the third hypothesis regarding whether visual and auditory phonological processing are overlapping.

**Figure 2.**
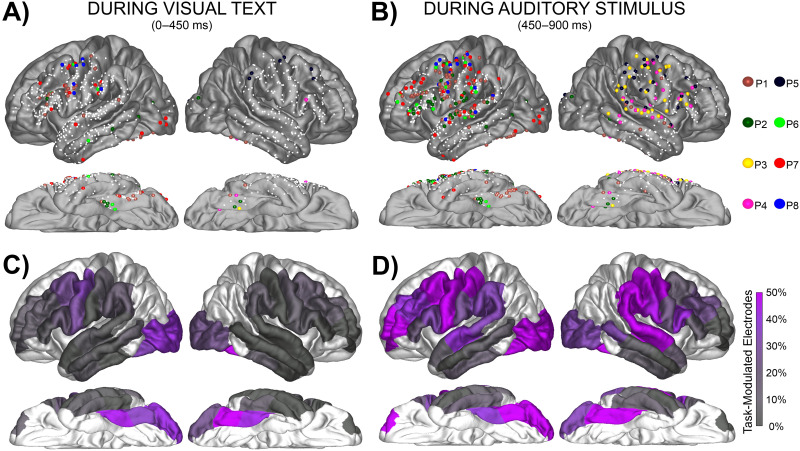
Task-Modulated electrode distribution across the cortex. (A–B) Electrodes demonstrating both a significant increase from baseline and a significant difference between the four trial types (Match, Mismatch, Visual Control, Auditory Control) during text presentation from 0–450 ms and during bi-phoneme presentation from 450–900 ms. Smaller white dots represent electrodes recorded from which did not meet criteria for being Task-Modulated (i.e., either did not show task-evoked activity or did not show modulation based on stimulus type). Each electrode is color-coded for individual patient. (C–D) Electrodes meeting criteria for a Task-Modulated effect displayed as percentages out of total electrodes in a ROI.

First we characterized the regions responding to and modulated by the match/mismatch task. Figure 1C–D displays the distribution and number of electrodes per parcellation. Supplementary Figure 1 displays electrode distribution color-coded by individual patient. (Supporting information can be found online at https://doi.org/10.1162/nol_a_00047). Figure 2 displays the distribution and proportions of Task-Modulated electrodes. Supplementary Figure 2 displays the proportions of Task-Modulated electrodes for each individual patient. Table 2 displays left hemisphere distribution of Task-Modulated electrodes. During the letter-string presentation, these effects (i.e., significant ANOVA differences between the four stimulus conditions as well as an overall increase above baseline) were overall left- (17%) versus right-lateralized (8%; Fisher’s Exact Test [FET]: *p* < 0.005). Activity was more widespread during bi-phoneme presentation and there was no left- (59%) versus right-lateralization (63%; FET: *p* > 0.05). Due to the left-lateralization of the reading network (Carreiras et al., 2014; Fiez & Petersen, 1998; Jobard et al., 2003; Price, 2012; Taylor et al., 2013) and the left-lateralized results during letter-string presentation, we will be focusing on left hemisphere comparisons moving forward.

**Table 2.** Distribution of Task-Modulated electrodes in each left-hemisphere region during letter-string presentation (0–450 ms) and bi-phoneme presentation (450–900 ms)

**Region**	**Left hemisphere**			
	**Task-Modulated (0–450 ms)**	**Subjects**	**Task-Modulated (450–900 ms)**	**Subjects**
Lateral occipital	42% (5 / 12)	3 / 3	50% (6 / 12)	3 / 3
Fusiform	39% (15 / 38)	4 / 4	50% (19 / 38)	3 / 4

Precentral	31% (17 / 54)	3 / 5	70% (38 / 54)	5 / 5
Postcentral	4% (2 / 50)	1 / 5	40% (20 / 50)	4 / 5

MTG	5% (2 / 43)	1 / 5	7% (3 / 43)	2 / 5
STG	4% (3 / 74)	2 / 5	38% (28 / 74)	5 / 5
Supramarginal	15% (6 / 41)	4 / 5	34% (14 / 41)	4 / 5

Pars opercularis	21% (8 / 39)	5 / 5	33% (13 / 39)	4 / 5
Pars triangularis	5% (2 / 38)	1 / 5	21% (8 / 38)	4 / 5
Middle frontal	12% (5 / 43)	2 / 5	40% (17 / 43)	3 / 5

*Note*. Task-Modulated columns: #% (#/#) = proportion of electrodes with Task-Modulated effect (electrodes showing effect / total electrodes). Subjects columns: # / # = number of patients with ≥1 electrode showing effect in region / total patients with electrodes in region.

Related to our first hypothesis that this GPC task would evoke at least as much activity in the precentral as the surrounding perisylvian regions, we found that during letter-string presentation, the left precentral gyrus had at least as great a proportion of Task-Modulated electrodes as the other temporal-parietal parcellations. The precentral parcellation had a greater proportion of Task-Modulated electrodes during letter-string presentation (31%, 4 of 5 patients) compared to the STG (4%, 2 of 5 patients; FET: *p* < 0.001) and not a significantly different proportion than the supramarginal (15%, 4 of 5 patients; FET: *p* < 0.05) or the pars opercularis (21%, 5 of 5 patients; FET: *p* > 0.05). In preparation for characterizing overlap (third hypothesis), we identified many electrodes during bi-phoneme presentation which demonstrated Task-Modulated effects. During bi-phoneme presentation, the precentral gyrus (70%, 5 of 5 patients) had a greater proportion of Task-Modulated electrodes than the STG (38%, 5 of 5 patients; FET: *p* < 0.001), supramarginal (34%, 4 of 5 patients; FET: *p* < 0.001), and pars opercularis (33%, 4 of 5 patients; FET: *p* < 0.001). Because proportion of electrodes displaying an effect is just one way to compare activity across regions, we also include Supplementary Figure 3, which displays the max BHG amplitudes for Task-Modulated electrodes across regions.

### Language-Preference Effects

**Figure 3.**
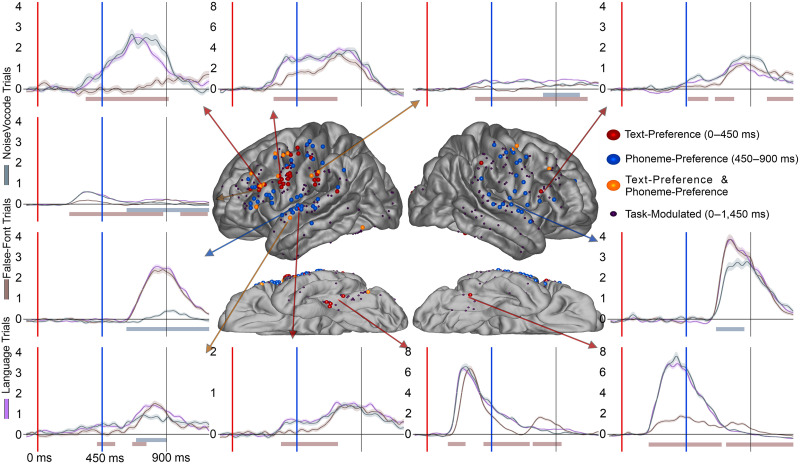
Text-Preference and Phoneme-Preference electrode distribution across the cortex. Display of electrode location (approximate, morphed to an average brain for display purposes) for Text-Preference (red), Phoneme-Preference (blue), and both effects (orange) electrodes. Smaller electrodes represent sites that were Task-Modulated (dark purple) but did not prefer language stimuli to controls. Arrows from specific electrodes are color-coded for the electrodes effect and point to examples illustrating typical waveforms for each region. Shaded regions surrounding the average waveforms reflect the standard error of the mean of the averaged trials. Vertical axis for BHG is in arbitrary units. The red shaded region at plot bottom highlights a significant Text-Preference effect period (letter-string > false-font) and the bluish bar highlights a significant Phoneme-Preference effect period (bi-phoneme > noise-vocoded). Analyses were temporally corrected using a bootstrapped shuffling of trial identity 1,000 times.

**Figure 4.**
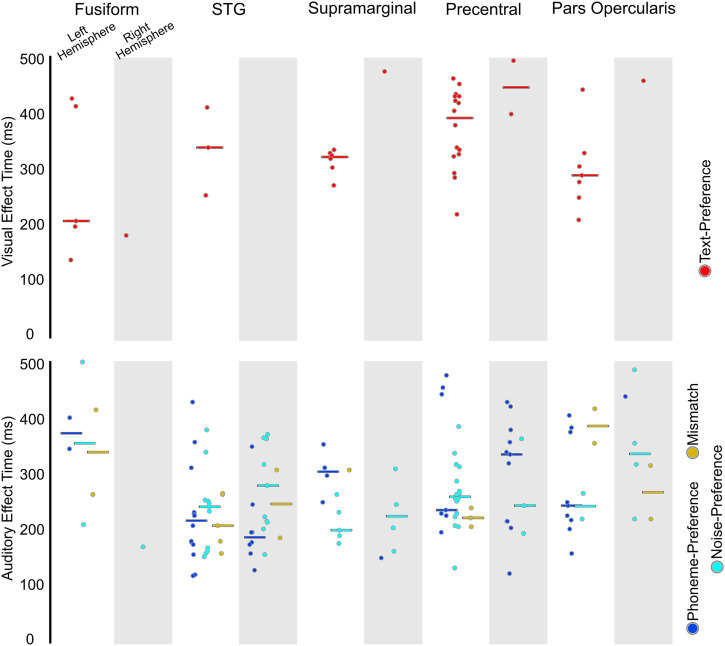
Effect onset timing from the letter-string presentation (top) and bi-phoneme presentation (bottom). Circles represent the onset of a significant effect at an individual electrode within each region. The top panel displays onset of effects from the start of letter-string presentation (i.e., starting from 0 ms) for Text-Preference (red) for the left hemisphere (not shaded) and right hemisphere (shaded). The bottom panel displays onset of effects from bi-phoneme onset (i.e., starting from 450 ms) for Phoneme-Preference (blue), Noise-Preference (cyan), and Mismatch effects (yellow) for the left hemisphere (not shaded) and right hemisphere (shaded).

Having established the strong modulation of the activity in the precentral parcellation by task stimuli, next we characterized which stimuli evoked responses in the precentral (Figure 3). Table 3 displays the distribution electrodes displaying effects. During the letter-string presentation we examined the Text-Preference effect (i.e., BHG to letter-string significantly greater than to false fonts) to understand if these effects were at least as consistently found in the precentral gyrus as in other perisylvian regions (hypothesis 1) and in a time window consistent with GPC (hypothesis 2). In the a priori left-hemisphere comparison, the precentral parcellation had more Text-Preference electrodes (30%, 4 of 5 patients) than the STG (4%, 2 of 5 patients; FET: *p* < 0.001) and was not significantly different from the supramarginal (15%, 4 of 5 patients; FET: *p* < 0.05) or pars opercularis (18%, 4 of 5 patients; FET: *p* > 0.05). For effect onset timing (Figure 4 and Table 4), there were no significant differences in Text-Preference onsets between the precentral gyrus (Earliest (1st): ∼220 ms; *Mdn*: ∼390 ms) and the pars opercularis (1st: ∼200 ms; *Mdn*: ∼280 ms; RankSum (Rs): *p* > 0.05), supramarginal (1st: ∼260 ms; *Mdn*: ∼320 ms; Rs: *p* > 0.05), or STG (1st: ∼240 ms; *Mdn*: ∼340 ms; Rs: *p* > 0.05). Therefore, there was consistent timing across regions during the time period associated with GPC, with all regions demonstrating effects during the GPC relevant time period.

**Table 3.** Distribution of electrodes displaying each effect divided into region in the left hemisphere

**Region**	**Selective electrodes**	**Text-Selective**	**Letter-Sensitive**	**Voice Selective**	**Noise-Vocoded Selective**	**Phoneme-Sensitive**	**Incongruent effects**
Lateral occipital	9	0	1	0	1	0	0
Fusiform	24	5	1	1	1	2	2

Precentral	39	16	3	7	16	8	3
Postcentral	25	1	0	8	5	10	0

MTG	5	0	0	1	0	1	0
STG	30	3	0	13	10	14	5
Supramarginal	19	6	0	4	5	2	1

Pars opercularis	19	7	1	9	2	0	2
Pars triangularis	12	1	0	5	0	1	1
Middle frontal	24	4	0	6	1	0	1

*Note*. # = number of electrodes in each region displaying the relevant effect.

**Table 4.** Median and range of regional effect onsets in the left hemisphere

**Region**	*Onset from text presentation (0 ms)*		*Onset from auditory presentation (450 ms)*			
	**Text-Selective**	**Letter-Sensitive**	**Voice-Selective**	**Vocode-Preference**	**Phoneme-Sensitive**	**Incongruent**
Fusiform	200 ms (140–420)	180 ms (180)	370 ms (340–400)	360 ms (220–500)	440 ms (440–440)	340 ms (260–420)
Lateral occipital	–	260 ms (260)	–	440 ms (440)	–	–
MTG	–	–	200 ms (200)	–	200 ms (200)	–
STG	340 ms (240–400)	–	220 ms (120–420)	240 ms (160–380)	180 ms (120–440)	220 ms (160–260)
Supramarginal	320 ms (260–320)	–	310 ms (260–360)	200 ms (180–260)	260 ms (180–340)	300 ms (300)
Precentral	390 ms (220–460)	420 ms (340–420)	240 ms (200–480)	260 ms (140–380)	280 ms (140–360)	220 ms (220–240)
Pars triangularis	420 ms (420)	–	280 ms (180–380)	–	300 ms (300)	380 ms (380)
Pars operculatris	280 ms (200–440)	220 ms (220)	240 ms (160–400)	240 ms (220–260)	–	390 ms (360–420)

*Note*. #ms (#–#) = median effect onset in ms (earliest effect onset in ms − latest effect onset in ms).

Here we also report Phoneme-Preference electrodes identified during the bi-phoneme presentation, which will be important to later considerations of overlap (hypothesis 3; explored in the Results section, Overlap of Text-Preference Electrodes With Auditory Effects). In the left-sided a priori comparisons, there were no significant differences between the precentral (13%, 4 of 5 patients) and the STG (18%, 3 of 5 patients; FET: *p* > 0.05), supramarginal (11%, 1 of 5 patients; FET: *p* > 0.05), or pars opercularis (23%, 4 of 5 patients; FET: *p* > 0.05) parcellations. Supplementary Figure 2 displays the proportions of Text-Preference and Phoneme-Preference electrodes for each individual patient.

### Letter- & Phoneme-Sensitive Effects

**Figure 5.**
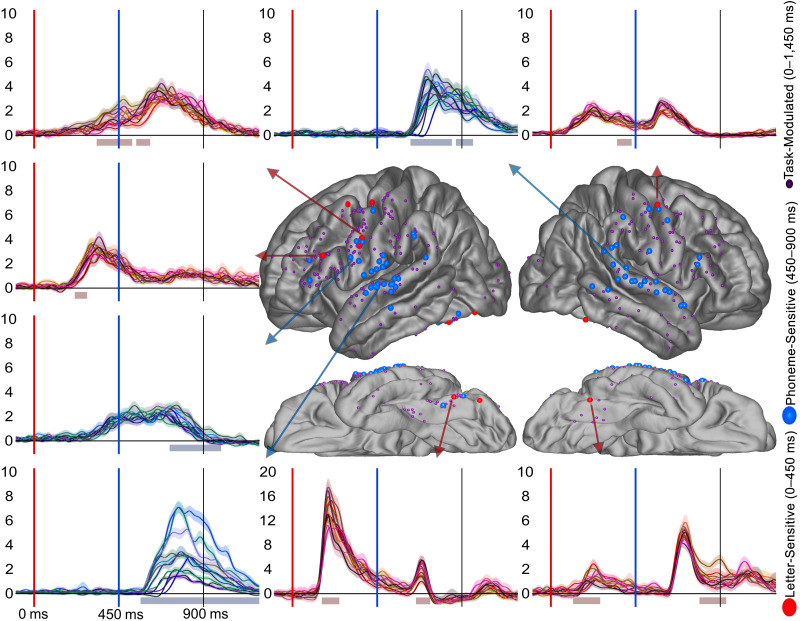
Letter-Sensitive and Phoneme-Sensitive electrode distribution across the cortex. Display of electrode location (approximate, morphed to an average brain for display purposes) for electrodes differentially responsive to individual letters (red) or phonemes (blue). Smaller electrodes represent sites that were Task-Modulated (dark purple) but did not demonstrate letter or phoneme identity sensitivity. Arrows from specific electrodes are color-coded for the electrode’s effect and point to examples illustrating typical waveforms for each region. Shaded regions surrounding the average waveforms reflect the standard error of the mean of the averaged trials. Each different line color represents the average response to one of the 12 consonant letters (reddish colors) or 12 consonant phonemes (bluish colors). Vertical axis for BHG is in arbitrary units. The red bar at plot bottom highlights a significant Letter-Sensitivity effect period and the blue bar highlights a significant Phoneme-Sensitivity effect period. Analyses were temporally corrected using a bootstrapped shuffling of trial identity 1,000 times.

Next, we sought to determine if there were any electrodes which responded differentially based on letter identity (i.e., Letter-Sensitive electrodes) and what the distribution of the electrodes was (hypothesis 1) and the timing of these effects (hypothesis 2). There were 8 Letter-Sensitive electrodes found. Though sparse, electrodes with effects were in expected ventral visual regions but more surprisingly also in frontal regions (Figure 5). The electrodes were in the ventral occipital-temporal regions (1 in the left lateral occipital, 1 in the left caudal fusiform, and 1 in the right caudal fusiform) and the lateral frontal (3 in the left precentral gyrus across 3 of the 5 patients; 1 in the left pars opercularis). While the greatest number of Letter-Sensitive electrodes were in the precentral parcellation it must be noted that in terms of proportion, the greatest proportion of Letter-Sensitive electrodes were found in the occipital-temporal regions as would be expected. Due to the limited number of electrodes, regional statistical comparisons were not informative. The lack of Letter-Sensitive effects is not surprising as the main region implicated in graphemic encoding, the ventral visual pathway centered on the posterior fusiform, was not well covered in this study. The earliest Letter-Sensitive effect was in the left caudal fusiform at ∼180 ms. The precentral Letter-Sensitive electrodes had a qualitatively later onset time (1st: ∼340 ms; *Mdn*: ∼420 ms). This further demonstrates the presence of reading-related effects in the precentral gyrus at a timing qualitatively a bit later than Text-Preference effects, but still during the letter-string presentation.

We next sought to understand the overlap of these Letter-Sensitive electrodes with Phoneme-Sensitive electrodes (hypothesis 3). Phoneme-Sensitive electrodes were more numerous, totaling 35 electrodes, with the highest proportion in STG. There were significantly more Phoneme-Sensitive electrodes in the precentral (15%, 3 of 5 patients) than the pars opercularis (0%, 0 of 5 patients; *p* = 0.01) but no difference between the precentral gyrus and the STG (19%, 3 of 5 patients; *p* > 0.05) or supramarginal (5%, 1 of 5 patients; *p* > 0.05). For the overlap, we note that the only region with both a Letter-Sensitive and a Phoneme-Sensitive effect in the same electrode was the precentral gyrus, though this was observed in only a single electrode.

### Mismatch Effects

**Figure 6.**
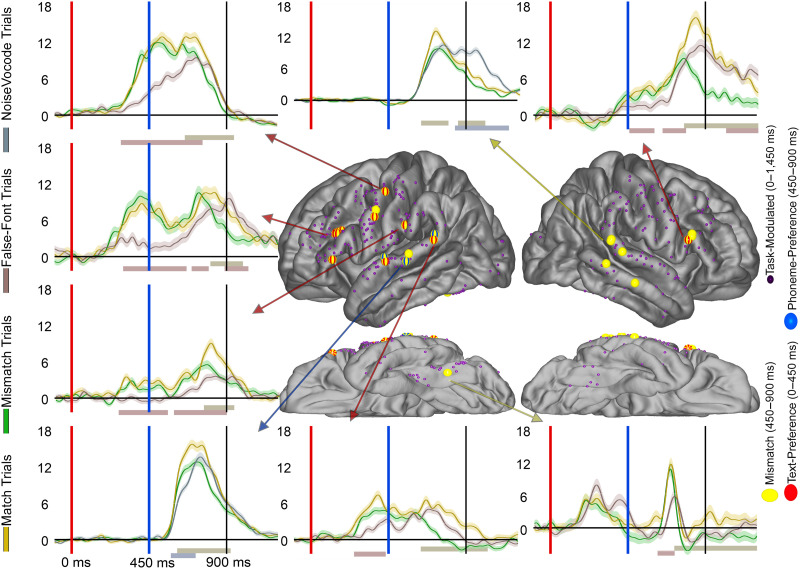
Mismatch effect electrode distribution across the cortex. Electrodes displayed on the brain for the Mismatch effect (yellow) across the cortex. Overlapping Text-Preference (red) and Phoneme-Preference (blue) effects are noted with stripes. Smaller electrodes represent sites that were Task-Modulated (dark purple) but did not demonstrate a Mismatch effect. Arrows from specific electrodes are color-coded for the electrodes effect and point to illustrating typical waveforms for each region. Shaded regions surrounding the average waveforms reflect the standard error of the mean of the averaged trials. Vertical axis for BHG is in arbitrary units. The red bar at plot bottom highlights a significant Letter-Preference effect period, the blue bar highlights a significant Phoneme-Preference effect period, and the yellow bar highlights a significant Mismatch effect period. Analyses were temporally corrected using a bootstrapped shuffling of trial identity 1,000 times.

Next, we focused on activity during the bi-phoneme presentation to understand the distribution of electrodes which showed evidence of cross-modal phonological priming. These effects could be evidence of overlapping visually-encoded and auditorily-encoded phonological representations (hypothesis 3). However, Mismatch effects were sparse, totaling 15 electrodes (Figure 6). These effects were concentrated mainly in the perisylvian regions of interest including the STG (5), precentral (3), pars opercularis (2), and supramarginal (1). Two electrodes with a Mismatch effect were found in the fusiform as well. The earliest Mismatch effect was in the STG at ∼160 ms post bi-phoneme presentation, with the median of the STG and precentral both occurring at ∼220 ms post bi-phoneme presentation. This was followed by the medians of the supramarginal (*Mdn*: ∼300 ms), fusiform (*Mdn*: 340 ms), and pars opercularis (*Mdn*: ∼390 ms).

### Overlap of Text-Preference Electrodes With Auditory Effects

**Figure 7.**
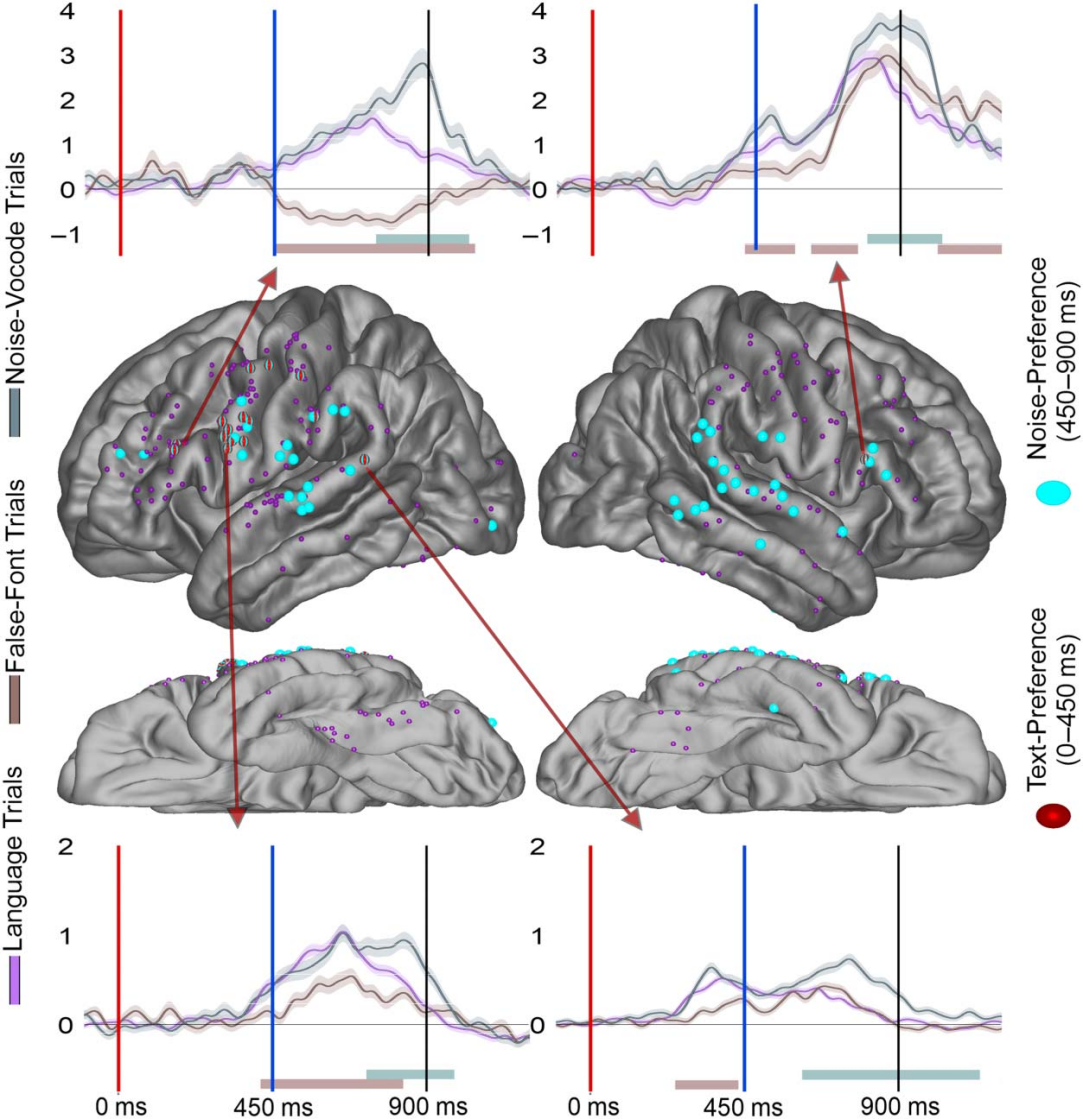
The overlap of Text-Preference and Noise- Preference effects. Display of electrode location (approximate, morphed to an average brain for display purposes) for Noise-Preference (cyan) electrodes. Preceding overlapping Text-Preference (red) effects are noted with red stripes. Smaller electrodes represent sites that were Task-Modulated (dark purple) but did not demonstrate a Noise-Preference effect. Arrows from specific electrodes point to an example typical waveform for each region. Shaded regions surrounding the average waveforms reflect the standard error of the mean of the averaged trials. Vertical axis for BHG is in arbitrary units. The red bar at plot bottom highlights a significant Letter-Preference effect period and the cyan bar highlights a significant Noise-Preference effect period. Analyses were temporally corrected using a bootstrapped shuffling of trial identity 1,000 times.

As a second assay into the relationship of visual language encoding to auditory language encoding (hypothesis 3), next we assessed overlap of visual and auditory language encoding by comparing Text-Preference electrodes’ overlap with three effects during the bi-phoneme presentation: Phoneme-Preference electrodes (Figure 3), Mismatch electrodes (Figure 6), and Noise-Preference electrodes (Figure 7). Because overlap within a region could happen by chance, we took the proportion of Text-Selective electrodes within a parcellation region as the baseline and compared this to the observed proportion.

For Phoneme-Preference effect electrodes, there was a significant overlap of Text-Preference with Phoneme-Preference electrodes (observed overlap (O): 25%, expected overlap (E): 10%; binomial test (BT): *p* < 0.001). However, at the regional level none of the perisylvian regions reached significance, though all showed numerically greater overlap than would be expected by chance: precentral (O: 43%, E: 30%; BT: *p* > 0.05), STG (O: 8%, E: 4%; BT: *p* > 0.05), supramarginal (O: 44%, E: 15%; BT: *p* > 0.05), and pars opercularis (O: 50%, E: 18%; BT: *p* > 0.05). For Mismatch effect electrodes, there was a significant overlap of Text-Preference and Mismatch electrodes (O: 37%, E: 10%; BT: *p* < 0.001). However, again no individual parcellation reached significance; the precentral parcellation (O: 20%, E: 31%; BT: *p* > 0.05), STG (O: 33%, E: 4%; BT: *p* < 0.05), and the pars opercularis (O: 100%, E: 18%; BT: *p* < 0.05) were all non-significant. For Noise-Preference electrodes, there was a significant overlap of Text-Preference and Noise-Preference electrodes (O: 37%, E: 10%; BT: *p* < 0.001; Figure 7). The perisylvian follow-up analyses identified the Precentral as having a significant relationship (O: 63%, E: 31%; BT: *p* = 0.01) but not the STG (O: 10%, E: 4%; BT: *p* > 0.05), supramarginal (O: 20%, E: 15%; BT: *p* > 0.05), or pars opercularis (O: 50%, E: 18%; BT: *p* > 0.05). Taken together, Text-Preference electrodes showed significantly greater overlap with auditory effects than would be predicted by chance across perisylvian regions, though in individual regions this rarely reached significance. We note that while the Text-Preference overlap was significantly greater than chance, the proportion of total overlap across the 3 auditory effects (25–37%) demonstrated that many electrodes showed only Text-Preference or only auditory effects.

### Connectivity Results

**Figure 8.**
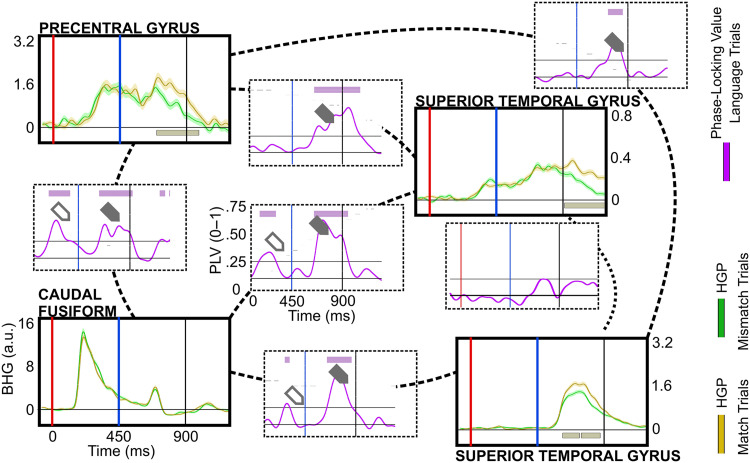
Illustration of a proposed grapheme-to-phoneme reading network in 4 electrodes from patient P1. Plots of waveforms in solid-line boxes illustrate broadband high gamma differences between Match (green) and Mismatch (yellow) trials. Shaded regions surrounding the average waveforms reflect the standard error of the mean of the averaged trials. The yellow bar at the plot bottom signifies periods of significant Mismatch effect. Analyses were temporally corrected using a bootstrapped shuffling of trial identity 1,000 times. Plots in dotted-line boxes illustrate phase-locking values (PLV) over time between electrodes for letter-string (purple) trials with the grey line the significance threshold for PLV for this subject, and the purple bar at the top showing periods of significant PLV. The open arrow highlights significant PLV during letter-string presentation; the filled arrow signifies significant PLV during bi-phoneme presentation. The red line at 0 ms denotes letter-string onset and the blue line at 450 ms denotes bi-phoneme onset.

Finally, we probed the possible networks involving the dorsal route using PLV (P1’s PLV results are displayed in Figure 8). During letter-string presentation, we characterized the distribution (hypothesis 1) and timing (hypothesis 2) of connectivity across two subnetworks: (1) a caudal fusiform to perisylvian subnetwork to capture connectivity between the ventral and dorsal reading routes, and (2) a precentral gyrus to perisylvian subnetwork. If the precentral gyrus is involved in GPC during reading, then its pattern of connectivity and the timing of this activity should include a relationship with either the fusiform (direct connection from the ventral reading route to the precentral) or from surrounding perisylvian regions (indirect connection from the ventral reading route to the precentral) during letter-string presentation.

For the caudal fusiform to perisylvian subnetwork during the letter-string presentation, 2 patients had a Task-Modulated electrode in the left caudal fusiform. Both patients had a ∼180 ms onset of Text-Preference effect, a timing associated with both fusiform orthographic processing (Hirshorn et al., 2016; Thesen et al., 2012) and widespread processing across the cortex (Halgren, 1990). The timing and location of these Text-Preference effects mark both patients’ electrodes as excellent assays into the temporal dynamics of the caudal fusiform orthographic hub’s relationship with the dorsal route. During letter-string presentation both electrodes showed significant PLV with precentral electrodes (P1 PLV-onset: ∼200 ms; P7 PLV-onset: ∼180 ms) and with an electrode in the supramarginal gyrus (P1: ∼400 ms; P7: ∼200 ms). But only 1 patient had significant PLV between the caudal fusiform and the STG (P1: ∼180 ms) and pars opercularis (P1: ∼180 ms). These patients’ BHG and PLV data are illustrated side-by-side in Supplementary Figure 4.

For the precentral to perisylvian subnetwork, 4 patients had Task-Modulated effects in the left precentral parcellation. These 4 patients displayed a muted connectivity between precentral electrodes and surrounding perisylvian regions during letter-string presentation, with only 1 patient displaying late significant PLV between the precentral and an electrode in the pars opercularis, (P1: ∼380 ms) and an electrode in the STG (P1: ∼420 ms). No patients had electrodes which displayed significant PLV between the precentral and supramarginal. Therefore, there is evidence of a direct connection between the ventral reading route and the precentral gyrus during reading, at a timing consistent with the onset of phonological processing in the dorsal perisylvian reading network.

Next, we examined the pattern of connectivity between perisylvian regions during the bi-phoneme presentation to understand how this functional network evolved when processing both visually and auditorily encoded phonemes (hypothesis 3). During the bi-phoneme presentation the precentral electrodes displayed more widespread PLV with surrounding perisylvian electrodes than during letter-string presentation. There were 3 out of 4 patients with significant PLV between electrodes in the precentral and STG (P1: ∼80 ms; P2: ∼180 ms; P7: ∼60 ms) and between the electrodes in the precentral and supramarginal (P1: ∼60 ms; P7: ∼60 ms; P8: ∼220 ms). Between the precentral and pars opercularis, 2 out of the 4 patients displayed significant PLV (P1: ∼160 ms; P2: ∼160 ms).

## DISCUSSION

Though theories of GPC have implicated articulatory phonemes, the place of the precentral gyrus in neuroanatomical models of reading is ambiguous (Carreiras et al., 2014; Fiez & Petersen, 1998; Taylor et al., 2013). Here we present evidence from a bimodal phonological match/mismatch task supporting a role for the precentral gyrus in mediating visual and auditory phonology. The presence of Text-Preference, Letter-Sensitive, and Mismatch effects occurred at rates at least as frequently in the precentral gyrus compared to other perisylvian regions cited by neurobiological models as involved in GPC. Further, the precentral had significant connectivity with the caudal fusiform during letter-string presentation and significant connectivity with adjoining temporal-parietal regions during bi-phoneme presentation. Further, the timing of the visual language evoked activity and connectivity in the precentral gyrus is consistent with the time window associated with GPC from extracranial electrophysiological research. The relationship of visual and auditory language evoked activity was mixed. Though there was significant overlap in evoked activity, there were also many electrodes in the perisylvian regions which were responsive to only a single language modality. Overall, our study is consistent with a role for the precentral gyrus in GPC.

### The Presence of Visual Language Processing in the Precentral Gyrus

The precentral gyrus’ relationship to articulatory phonological representations and STG’s relationship to encoding phonological representations is important to long-standing debates regarding the relative contributions of articulatory versus encoding phonemic representations to silent reading (Besner, 1987; Frost, 1998). fMRI studies have reported evoked BOLD activation during reading tasks in the STG and other temporal-parietal regions (Booth, 2002; Rumsey et al., 1997; Simos et al., 2002) as well as the precentral gyrus and inferior frontal regions (Binder et al., 2005; Dehaene et al., 2001; Fiez et al., 1999; Price et al., 1997; Yen et al., 2019). We replicate these studies, with a significant proportion of precentral gyrus electrodes (30%) showing Text-Preference effects, as well as Text-Preference effects in the STG (4%), supramarginal (15%), and pars opercularis (18%). In addition, the precentral gyrus also had 3 electrodes across 3 patients in which individual Letter-Sensitive electrodes were found. Finally, in the 2 patients that had a Task-Modulated electrode in the left caudal fusiform, significant connectivity between the electrodes in the fusiform and electrodes in the precentral gyrus was found. Taken together, these findings offer strong replications of previous research findings of precentral involvement during reading, here during a GPC specific task.

The nature of visual phonological processing in the precentral gyrus will require additional study to better understand the possible functional response profiles. In this study, electrodes responding to visual text appeared spatially distributed in both the inferior (close to the inferior frontal sulcus) and more dorsal (close to the superior frontal sulcus) precentral gyrus. The inferior region is adjacent to the inferior frontal gyrus, and this larger region including the inferior precentral gyrus and pars opercularis has been reliably found to be more activated by phonological processing tasks rather than semantic processing tasks during reading (Gitelman et al., 2005; McDermott et al., 2003; Price et al., 1997). This inferior precentral region may be broadly overlapping with mouth articulatory movements and has been hypothesized to be involved in speech processing (Pulvermüller et al., 2006). However, evidence has also emerged of a more dorsal region in the precentral gyrus separate from articulatory areas that is also involved in speech perception (Berezutskaya et al., 2020; Cheung et al., 2016). The relationship of these two areas, the inferior and more dorsal precentral, during speech perception is still under study. In our study focusing on visual language, electrodes from both the more inferior and the more superior regions shared a similar response waveform during our task (Supplementary Figure 5). The timing of onsets to visual text, the onset and duration of Text-Preference effects, and the sustained nature of activity throughout the presentation of the bi-phoneme were consistent across both the inferior and the more dorsal regions. Future study will be necessary to understand how phonological representations across the precentral gyrus are encoded during reading.

### The Time-Course of the Precentral Gyrus’ Involvement in Visual Text Processing

The time-course of GPC is important to understanding the precentral gyrus’ potential role in silent reading. Visual word encoding proceeds along the ventral temporal reading route in a feedforward sweep (Dehaene & Cohen, 2011; Lochy et al., 2018; Vinckier et al., 2007). Visual processing begins at 60 ms in posterior visual cortex (Foxe & Simpson, 2002) with Letter-Sensitive encoding onsets in posterior occipital-temporal regions ∼160–180 ms (Allison et al., 1994, 1999; Hirshorn et al., 2016; Thesen et al., 2012). Lexical-semantic effects begin in the anterior-ventral temporal lobe at ∼200–250 ms (Chan et al., 2011; Lochy et al., 2018; Nobre et al., 1994; Nobre & McCarthy, 1995; Thesen et al., 2012). These lexical-semantic onset times align well with the onset of the rough time period from ∼250–500 ms associated with the N400 complex, taken to index lexical-semantic integration (Marinković, 2004). During this extended N400 complex time period, prolonged feedforward/feedback interaction in the language network has been observed (Halgren et al., 2006, 2015). In dorsal regions simultaneous and widespread activity begins at ∼180 ms (Halgren, 1990), and in the inferior frontal gyrus (Sahin et al., 2009), latencies consistent with Letter-Sensitive effects begin in caudal occipital-fusiform regions. It is during the widespread and simultaneous integrative period of feedforward/feedback interaction that GPC and phonemic integration from the dorsal regions would be expected.

During letter-string presentation we found two subjects with both a Text-Preference onset at ∼160 ms in the caudal fusiform and significant connectivity between caudal fusiform and dorsal route regions. This supports the caudal fusiform, strongly associated with orthographic processing (Cohen et al., 2000; Dehaene et al., 2001; Gaillard et al., 2006; Lüders et al., 1991), as a hub connecting the ventral reading route with the widespread dorsal reading route activity. Indeed, a resting-state fMRI study found the fusiform preferentially linked with frontal regions including the precentral and pars opercularis as well as posterior temporal-parietal regions (Stevens et al., 2017). These dorsal regions’ earliest Text-Preference effects were in pars opercularis at ∼200 ms (*Mdn*: 280 ms), precentral at ∼220 ms (*Mdn*: 390 ms), STG at ∼240 ms (*Mdn*: 340 ms), and supramarginal at ∼260 ms (*Mdn*: 320 ms) with no statistically significant differences in onset times. This pattern is consistent with widespread early and sustained processing in the dorsal (i.e., putative phonological) reading network during visual language encoding. Effects indexing individual letter identity sensitivity (i.e., Letter-Sensitive effects) emerged later in the precentral gyrus at ∼340 ms (*Mdn*: ∼420 ms) compared to earlier Letter-Sensitive effects in the caudal fusiform at ∼180 ms (*Mdn*: ∼230 ms), demonstrating quicker onsets in visual orthographic processing coupled with later dorsal processing.

This observed timing aligns with extracranial EEG studies investigating the timing of phonological priming. Pseudohomophone priming effects (e.g., “BRANE” primes “BRAIN”) are found during evoked components starting at ∼250 ms (Grainger et al., 2006). Simultaneous presentation of visual and auditory words that either match or mismatch shows differences beginning at ∼300 ms (Holcomb & Anderson, 1993). Rhyme judgement evoked differences at ∼300–350 ms (Bentin et al., 1999; Rugg, 1984; Rugg & Barrett, 1987). These extracranial EEG studies provide evidence that phonological effects begin from ∼250–350 ms, depending on the paradigm used, the same time window as visual language evoked activity onset across our perisylvian dorsal (i.e., putative phonological) route.

### The Relationship Between Visual and Auditory Language Processing in Perisylvian Regions

Cognitive models of reading implicitly or explicitly theorize that GPC links orthographic processing to the existing auditory language system. Here we directly test this theory by examining the overlap and separation as well as the priming of phonological representations activated first by visual language input then second by auditory language input. The STG’s role in encoding auditorily presented phonemes (Leonard et al., 2015; Mesgarani et al., 2014; Travis et al., 2013) suggests that it may also help encode visually presented phonemes. Previous intracranial studies have found overlapping activation in the STG to visually and auditorily presented words (Perrone-Bertolotti et al., 2012) and individual neurons with correlated firing between phonemes and graphemes (Chan et al., 2014) and noted resting state connectivity between the STG and fusiform (Stevens et al., 2017). We confirm this role for the STG in phonemic encoding, with strong STG responsivity during the bi-phoneme presentation and many Phoneme-Preference and Phoneme-Sensitive effects. Previous research has also found involvement of the precentral gyrus in auditory language processing (Cheung et al., 2016), which we replicate here with the precentral gyrus showing Phoneme-Preference and Phoneme-Sensitive effects.

To understand if phonological processing during reading makes use of the existing auditory language system, we examined whether visually evoked effects overlapped with auditorily evoked effects and whether visually encoded phonemes prime the auditory encoding of phonemes. We did find significant overlap of Text-Preference with Phoneme-Preference electrodes. However, while this overlap was significantly greater than chance it was not comprehensive (25% of Text-Preference electrodes were also Phoneme-Preference), with many effects in perisylvian regions which were only evoked by visual language. Perhaps surprisingly, only limited evidence of visual phonological priming of auditory phonemes was found, evidenced by the electrodes with Mismatch Effects. If the exact same phonological representations in the perisylvian language system are activated by both visual and auditory language input, then widespread priming effects would be expected (Gotts et al., 2012). While we did find mismatch effects in the STG, precentral, pars opercularis, and supramarginal gyrus, they were overall sparse, totaling just 15 electrodes. The median onset time of Mismatch effects in both the precentral gyrus and STG was ∼220 ms post bi-phoneme presentation, which is right around the time the first phoneme of the bi-phoneme pair was complete and in line with timing in reports of visual/auditory priming in the extracranial EEG literature (Holcomb et al., 2005; Kiyonaga et al., 2007). The STG and precentral showed significant PLV during the bimodal period of comparison, evidence for integration of grapheme and phoneme identity. Indeed, during bi-phoneme presentation both regions displayed significant PLV with each. The earliest Mismatch effect was found in the STG at ∼160 ms, in a region which was completely unresponsive during the preceding letter-string presentation implying that this patch of STG was involved in the integration between grapheme and phoneme but not the processing of the grapheme. In total, the distribution of visual and auditory language effects suggests both overlapping and separate language representations for visual language processing in perisylvian cortex.

### Limitations

The data we present is from a small number of patients who had electrophysiology recorded directly from the cortical surface. Though this data is valuable for understanding cognitive function due to possessing high spatial and temporal precision, qualifications are necessary due to the rarity of this data and therefore the low number of patients included in this study. Of the 8 patients recorded from, 5 had implantations covering the left hemisphere. As GPC is a predominantly left-lateralized function and our patients were all presumed to have left-lateralized language, attention must be paid to our small sample size. Further, electrode placement is based on clinical rather than research considerations, and surface-based electrodes lack access to sulcal cortex. Therefore, it is difficult to make strong inferences about interregional comparisons. Further, though we found interesting effects, the limited number of electrodes necessitates further replication. For example, Letter-Sensitive electrodes we found in the precentral gyrus, a total of 3 electrodes in 3 patients. The Letter-Sensitive effects in the precentral gyrus differed in waveform, a sharp waveform in the fusiform versus a boxier elongated waveform in the precentral, and differed in timing, early (*Mdn*: ∼180 ms) for the fusiform and later (*Mdn*: ∼420 ms) for the precentral. However, it is not possible to conclude a unique role for the precentral gyrus in GPC from these electrodes but they do offer a target for further exploration. Finally, 3 subjects did not have definitive language lateralization available via Wada; however, these patients were all right-handed and therefore have a high likelihood of having left-lateralized language.

### Conclusion and Future Directions

Early psychological theories emphasized a place for subvocal articulation in silent reading (Allport, 1979; Barron & Baron, 1977; Burani et al., 1991; Klapp, 1971; Kleiman, 1975; Peterson & Johnson, 1971; Sun & Peperkamp, 2016). In the Self-Learning Hypothesis model, articulation is a key determinant in learning to read (Share, 1995). Articulating during reading facilitates learning (Cunningham et al., 2002; Kyte & Johnson, 2006) and motor cortex disruption in childhood by seizures in rolandic epilepsy causes long-term reading impairments (Clarke et al., 2007; Piccinelli et al., 2008; Staden et al., 1998). A neuroimaging review of reading disorders linked increased precentral cortex BOLD activation to compensatory mechanisms centered on increased reliance on GPC in disordered reading (Hancock et al., 2017). Computational modeling of learning to read (Harm & Seidenberg, 2004), as well as empirical evidence (Grainger et al., 2012), shows a shift from reliance on phonological to orthographic information as reading skill increases; however, peak performance requires both types of information (Harm & Seidenberg, 2004), and phonological information continues to influence silent reading (Frost, 1998; Perfetti et al., 1988; Rastle & Brysbaert, 2006). Therefore, we propose that the putative functional relationship between precentral gyrus and caudal fusiform suggested by this study is likely formed when articulating during learning to read and remains a contributing factor to silent reading throughout the lifespan. The empirical evidence presented here does not conclusively establish an articulatory mechanistic understanding of GPC. Indeed, auditory phonological processing in the more dorsal aspect of the precentral gyrus is reported to be related to acoustic rather than articulatory representations (Cheung et al., 2016), raising the possibility of multiple response profiles in precentral phonological processing. During reading, a finer spatial resolution is needed to identify cortical patches, and potentially individual neurons, representing overlapping letter/phoneme identities and overlapping encoding/articulation. The greatly increased spatial resolution permitted by next-generation electrodes (Ganji et al., 2017; Khodagholy et al., 2015) will allow for the measuring and stimulating down to a width of 50 μm to test the articulatory GPC hypothesis.

## ACKNOWLEDGMENTS

Supported by NIH R01 NS018741, Kavli Institute for Brain and Mind, and Chancellor’s Collaboratories Award (University of California San Diego).

## FUNDING INFORMATION

Eric Halgren, National Institute of Neurological Disorders and Stroke (https://dx.doi.org/10.13039/100000065), Award ID: NS018741.

## AUTHOR CONTRIBUTIONS

**Erik Kaestner**: Conceptualization; Project administration; Software; Formal analysis; Writing – original draft; Visualization. **Xiaojing Wu**: Conceptualization; Data curation; Investigation; Writing – review & editing. **Daniel Friedman**: Investigation; Writing – review & editing. **Patricia Dugan**: Investigation; Writing – review & editing. **Orrin Devinsky**: Investigation; Writing – review & editing. **Chad Carlson**: Investigation; Writing – review & editing. **Werner Doyle**: Investigation; Writing – review & editing. **Thomas Thesen**: Investigation; Writing – review & editing. **Eric Halgren**: Conceptualization; Project administration; Resources; Writing – original draft; Funding acquisition.

## TECHNICAL TERMS

Grapheme-to-phoneme conversion: The conversion from a visual orthographic code into an auditory phonological code during reading.
Broadband gamma power: Gives an indirect measure of the aggregate surrounding neuronal population firing, here calculated by measuring the power in the frequency range 70–170 Hz.
Phase-locking value: Measures the consistency of the relative phase of frequencies within the electrophysiological signal between two electrodes.
