# Supplementary material for: The Precentral Gyrus Contributions to the Early Time-Course of Grapheme-to-Phoneme Conversion
Source: Neurobiol Lang (Camb). 2022 Feb 10;3(1):18–45. doi: 10.1162/nol_a_00047 (PMC10158576; doi:10.1162/nol_a_00047)
Supplement: Supplementary file 1 [file nol-3-1-18-s001.docx]

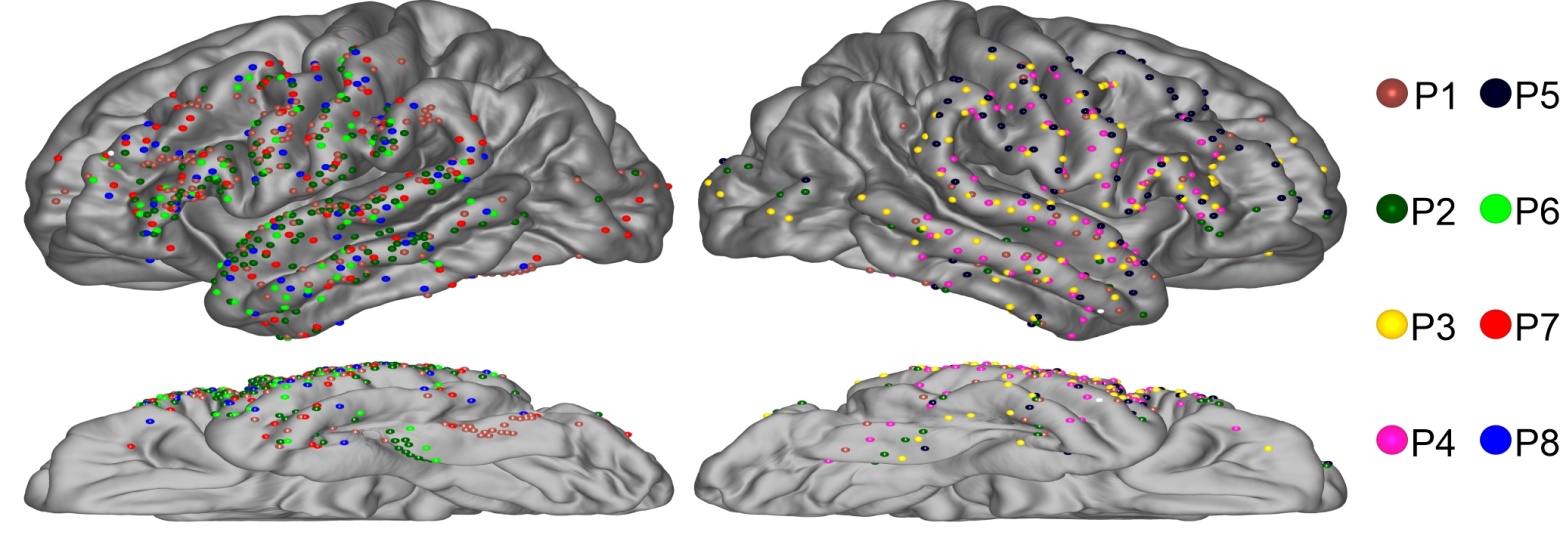


**Supplementary Figure 1. Electrode coverage color-coded by patient.** Electrode coverage highlighting coverage of perisylvian electrodes. Each individual color represents electrodes from a single patient.

**
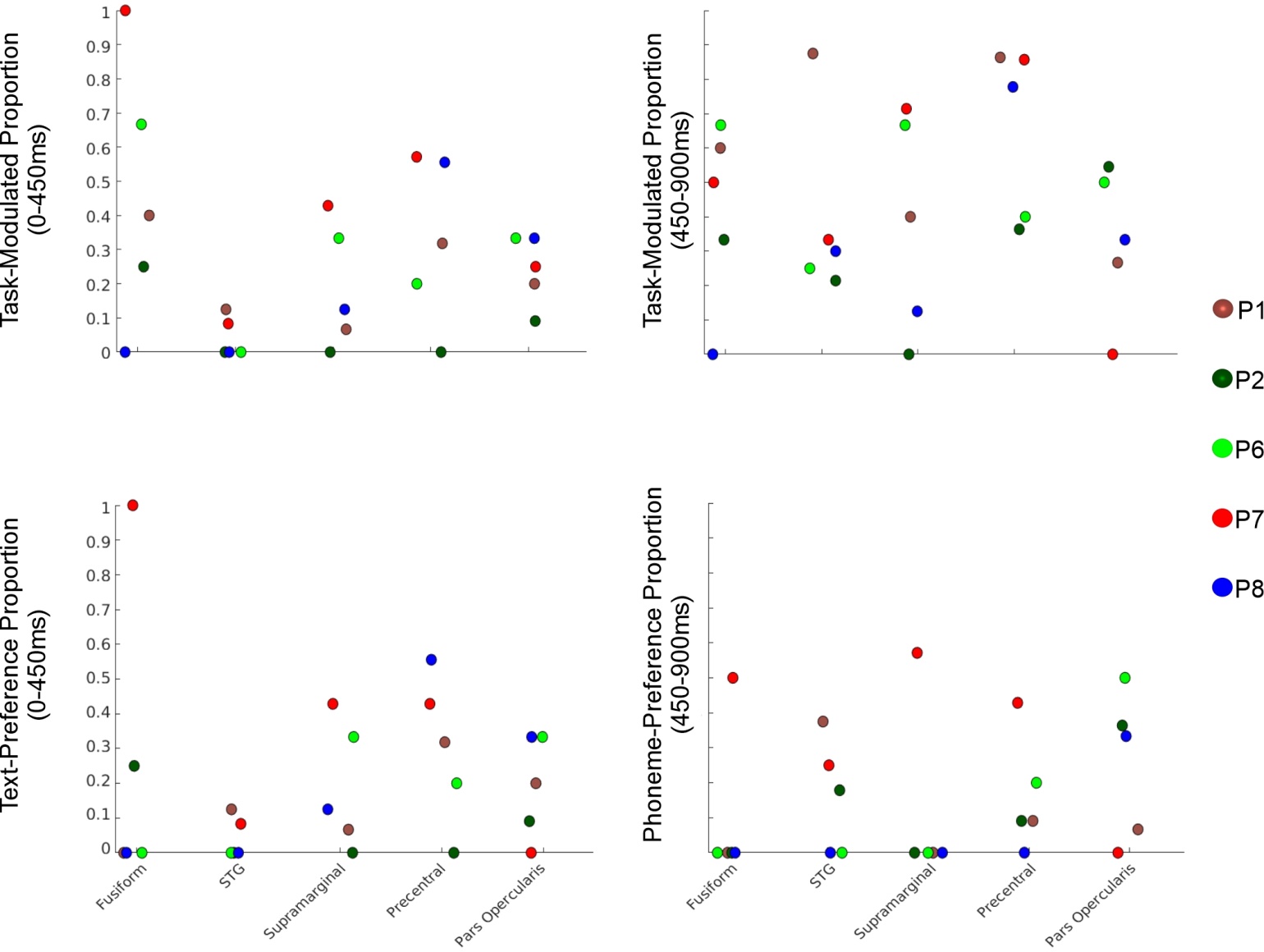
**

**Supplementary Figure 2. Proportions of left-hemisphere regional effects for each individual patient with a primarily left hemisphere implantation.** Each of the 5 patients with predominantly left-sided implantations have their individual proportions displayed for Task-Modulated effects during visual and auditory stimulus presentation, Text-Preference effects, and Phoneme-Preference effects across 5 perisylvian regions.

**
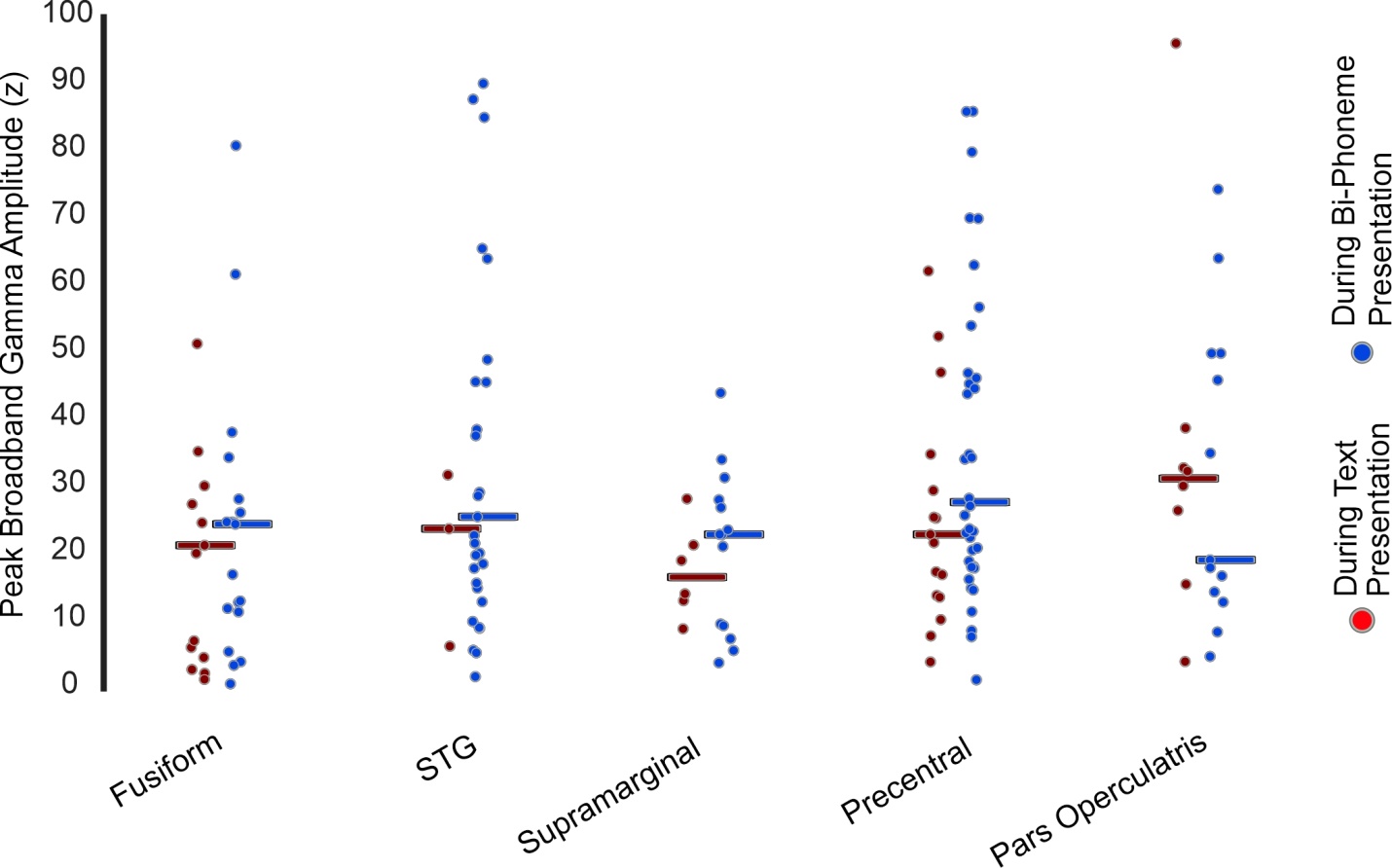
**

**Supplementary Figure 3. Peak broadband gamma amplitude during text presentation and during bi-phoneme presentation.** Circles represent the peak amplitude of broadband gamma amplitude of Task-Modulated electrodes from across all predominantly left hemisphere implantation patients in 5 left hemisphere regions. The red circles display peak amplitude during text presentation (0-450ms) while the blue circles display peak amplitude during bi-phoneme presentation (450-900ms). Colored bars represent median amplitudes for the region.

**
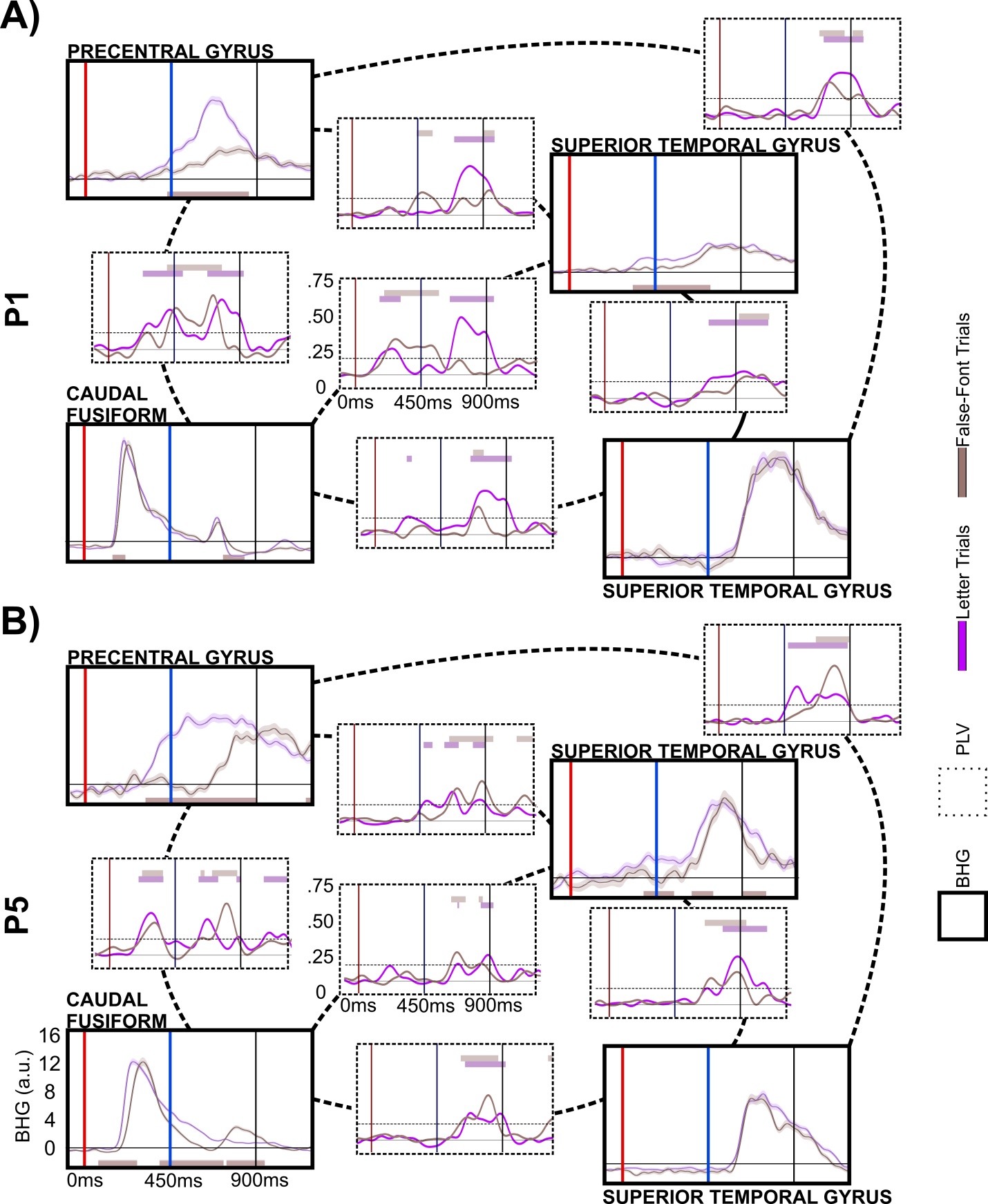
**

**Supplementary Figure 4. Illustration PLV compared between letter-string trials and false-font trials in electrodes from 4 regions in patients P1 and P5.** Plots of waveforms in solid lined boxes illustrate broadband high gamma differences between letter-string (purple) and false-font (reddish-grey) trials. Shaded regions surrounding the average waveforms reflect the standard error of the mean of the averaged trials. The reddish-grey bar at the plot bottom signifies periods of significant Letter-Preference effect. Analyses were temporally corrected using a bootstrapped shuffling of trial identity 1000 times. Plots in dotted-line boxes illustrate phase-locking values (PLV) over time between electrodes for letter-string (purple) and false-font (reddish-grey) trials with the grey line the significance threshold for PLV for this subject, and the bar at the top showing periods of significant PLV for letter-string (purple) and false-font (reddish grey) trials. The red line at 0ms denotes letter-string onset and the blue line at 450ms denotes bi-phoneme onset.

**
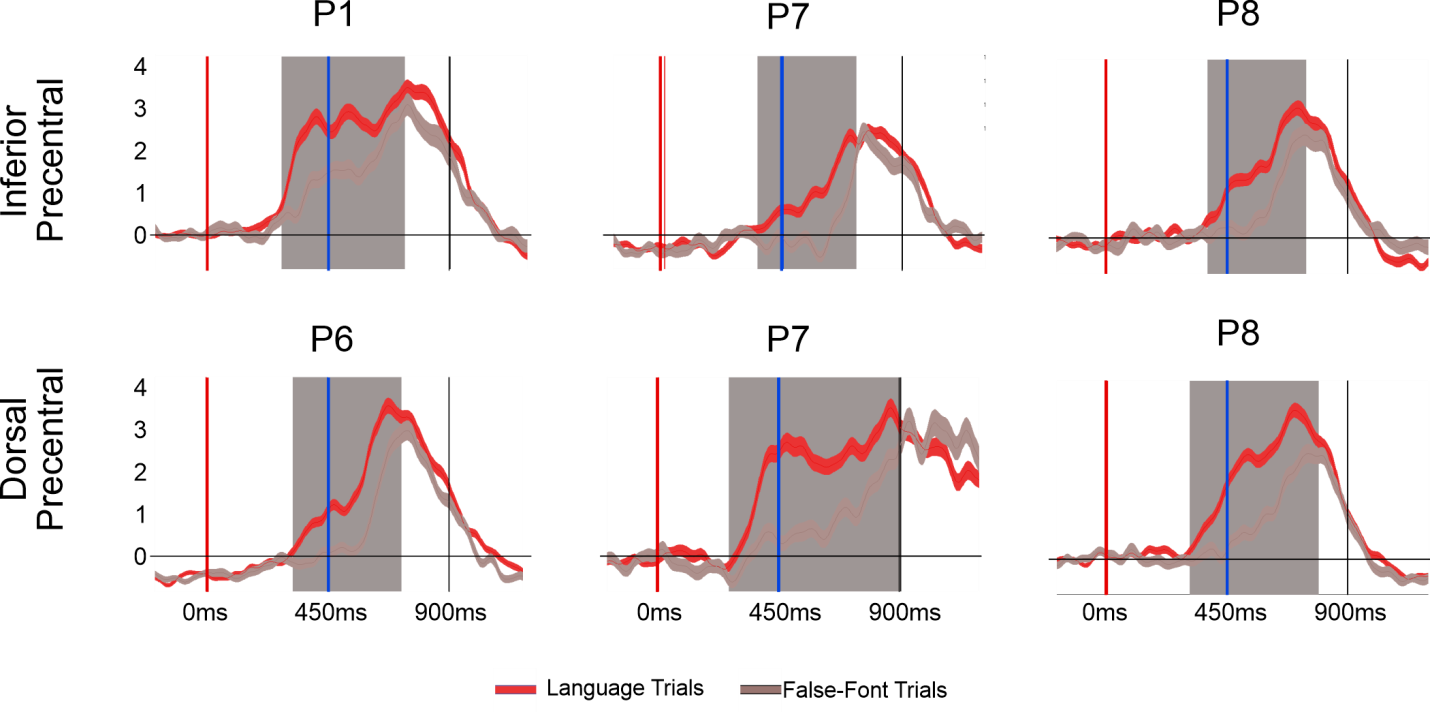
**

**Supplementary Figure 5. Illustrating the consistency of waveforms between Text-Preference effects in the inferior and dorsal precentral.** Shaded regions surrounding the average waveforms reflect the standard error of the mean of the averaged trials. Vertical axis for BHG is in arbitrary units (a.u.). The red shaded region at plot bottom highlights a significant Text-Preference effect period (letter-string > false-font). Analyses were temporally corrected using a bootstrapped shuffling of trial identity 1000 times. Dorsal and inferior parts of the precentral gyrus as defined by splitting the Desikan precentral ROI. P# = the patient the electrode came from.
